# Supplementary material for: Creating SPACE to evolve academic assessment
Source: eLife. 2021 Sep 23;10:e70929. doi: 10.7554/eLife.70929 (PMC8460251; doi:10.7554/eLife.70929)
Supplement: Supplementary file 1. — It was designed for universities and other institutions who want to reform how they assess research and researchers. [file elife-70929-supp1.pdf]

RETHINKING RESEARCH ASSESSMENT

S.P.A.C.E. TO EVOLVE ACADEMIC ASSESSMENT

A RUBRIC FOR ANALYZING INSTITUTIONAL PROGRESS INDICATORS AND CONDITIONS FOR SUCCESS

DORA

Research and researcher assessment is a systems challenge, suggesting that institutions that prioritize developing infrastructures to support their efforts may be better positioned to achieve their goals than those focused only on individual solutions.

	FROM **FOUNDATION...**<br>*Core definitions and shared clarity of purpose*	TO **EXPANSION...**<br>*Increased traction and capability development*	TO **SCALING**<br>*Accelerated uptake and continuous improvement*
**STANDARDS FOR SCHOLARSHIP**<br>How are new definitions of "quality scholarship" formulated and applied?	**ALIGNMENT** ON VALUES AND GOALS<br>*THIS MIGHT LOOK LIKE...*<br>Standards are explicitly **designed and articulated to align with institutional mission and values**, such as increasing equity and support for traditionally underrepresented, minoritized groups<br>New standards for scholarship consider the **balance across research, teaching, and service** contributions including training, mentoring and good citizenship<br>Specific definitions and standards of "quality" with regard to scholarship are articulated and **shared across disciplines** and review/promotion committees	**DIVERSIFICATION** OF STANDARDS<br>*THIS MIGHT LOOK LIKE...*<br>Scholarship is assessed using **diverse indicators** (e.g. societal impact), **units of assessment** (e.g. full body of work v. individual articles), and **forms of output** (e.g. non-journal contributions)<br>Indicators of quality recognize **non-individualized activities and accomplishments** like team science<br>New definitions of "scholarship" are **deployed across the full range of institutional disciplines**	**ADOPTION** OF NEW PRACTICES<br>*THIS MIGHT LOOK LIKE...*<br>Faculty have the ability to **customize success measures** to reflect their research interests and goals<br>New standards, definitions, and criteria for evaluating the quality and impact of scholarship are **integrated into the language and processes of new assessment practices**
**PROCESS MECHANICS AND POLICIES**<br>How are new practices incorporated into review structures, processes, and institutional policies?	**DEBIASING** DELIBERATIVE JUDGMENTS<br>*THIS MIGHT LOOK LIKE...*<br>Meaningful and appropriately **rigorous qualitative structures for academic assessment**, such as narrative CVs, are given due weight<br>Structures and processes are **applied consistently across assessment activities,** taking into consideration alternate paths and starting points<br>Use of **new assessment mechanics extend beyond traditional evaluative contexts** into ensuring equitable opportunities, mentoring, and retention to increase research and researcher diversity	**CAPACITY** TO SUPPORT NEW ACTIVITIES<br>*THIS MIGHT LOOK LIKE...*<br>**Training on the goals and procedures of assessment processes and practices** are accessible and continually maintained<br>Institutions design processes take into account the **resource capacity of committee members to effectively adopt new assessment practices**, such as additional burdens on time<br>Institutions have **designated senior functions or offices to ensure faculty capacity** for new assessment practices and principles	**INTEGRATION** INTO EXISTING SYSTEMS<br>*THIS MIGHT LOOK LIKE...*<br>Assessment mechanics can be **flexibly applied and adapted** to accommodate diverse disciplines<br>Mechanisms to support practices are **codified and written into institutional policies**<br>New processes and practices are **seamlessly integrated** and widely adopted
**ACCOUNTABILITY**<br>How are individuals and institutions held liable for executing on new assessment practices?	**TRANSPARENCY** AND CLARITY OF GOALS<br>*THIS MIGHT LOOK LIKE...*<br>The goals, principles, and practices of academic assessment and review, promotion, and tenure (RPT) activities are **transparent and clearly articulated,** and agreed upon by all participants<br>Institutions have **clearly defined expectations for adherence** to academic assessment practices<br>**Examples of "what good looks like"** are collected and shared to more concretely illustrate target outcomes and behaviors	**ADHERENCE** THROUGH COMMITMENT<br>*THIS MIGHT LOOK LIKE...*<br>Research evaluators **self-monitor adherence** to academic assessment principles and practices<br>**Senior leaders and committee members actively stipulate equitable assessment practices** during both formal and informal career development contexts<br>Institutions model **ecosystem-level accountability**, such as ensuring that system-level incentives align with and support agreed-upon principles and practices	**PROACTIVITY** IN ENGAGEMENT<br>*THIS MIGHT LOOK LIKE...*<br>**Individuals actively contribute** to the development and review of new practices and principles<br>**Departments proactively broaden and conduct outreach activities** to include new or minoritized applicants<br>**Faculty serve as "ambassadors"** for new academic assessment practices, such as when serving as external committee members
**CULTURE WITHIN INSTITUTIONS**<br>How are assessment practices perceived and adopted both within and outside of formal evaluation activities?	**INCLUSION** AND ACCESS<br>*THIS MIGHT LOOK LIKE...*<br>More **diverse types of individuals are involved in both defining and participating in career advancement processes**, such as including early career researchers on RPT committees<br>**Representation of minoritized applicants meets or exceeds equity goals** for both new hires and researcher retention<br>Career growth and mentoring systems are intentionally designed to provide **ongoing support for underreprsented hires**	**ADVOCACY** AT INSTITUTIONAL LEVELS<br>*THIS MIGHT LOOK LIKE...*<br>Adoption of new assessment mechanisms is **supported and advocated for by departmental and institutional leaders**<br>**All individuals actively contribute** to building more equitable practices—not just minoritized ones<br>New **research assessment norms are increasingly adopted as a default** by faculty, administrators, and applicants	**REFLEXIVITY** THROUGH REFLECTION<br>*THIS MIGHT LOOK LIKE...*<br>"Positive friction," or intentional **pause points to reflect on assessment practices and slow down business-as-usual processes** is incorporated into both formal and informal assessment practices<br>All participants in assessment activities feel processes achieve a **balance of effectiveness and efficiency**
**EVALUATIVE AND ITERATIVE FEEDBACK**<br>How are intervention outcomes and progress toward institutional values captured and continually improved upon?	**ARTICULATION** OF DIVERSE INDICATORS<br>*THIS MIGHT LOOK LIKE...*<br>**Goals and success criteria** for individual academic assessment interventions are well-defined and shared<br>Use of **leading indicators** (e.g. increased diversity of inquiries for open positions) supplements lagging indicators (e.g. increased diversity of hires) when gauging intervention efficacy<br>Goals and **success criteria are automatically reviewed** whenever institutional strategy is updated	**SYSTEMATIZATION** TO GAIN CONSISTENCY<br>*THIS MIGHT LOOK LIKE...*<br>Quantitative and qualitative **data from interventions are captured in a standardized way**<br>Mechanisms that **capture both quantitative and qualitative feedback** are explicitly designed and embedded into assessment processes from the outset<br>**Best practices and examples** of measurement and/or gathering feedback are codified and shared across disciplines within the institution	**IMPROVEMENT** USING FEEDBACK LOOPS<br>*THIS MIGHT LOOK LIKE...*<br>Interventions that don't achieve desired outcomes are considered **learning opportunities**, not failures<br>Outcomes and data are collected and monitored to ensure high standards of evaluation quality and identify **unintended consequences or adverse effects**<br>Feedback and other **indicators are refined and/or examined in aggregate** to identify and investigate patterns or opportunities for course-correction

This is Open Access content distributed in accordance with the Creative Commons Attribution 4.0 International License

Hatch, A and R. Schmidt. (2021) Rethinking Research Assessment: SPACE to Evolve Academic Assessment. DORA.

RETHINKING RESEARCH ASSESSMENT

S.P.A.C.E. TO EVOLVE ACADEMIC ASSESSMENT

A RUBRIC FOR ANALYZING INSTITUTIONAL PROGRESS INDICATORS AND CONDITIONS FOR SUCCESS

	FOUNDATION	EXPANSION	SCALING
STANDARDS FOR SCHOLARSHIP	**Alignment** on values and goals	**Diversification** of standards	**Adoption** of new practices
PROCESS MECHANICS AND POLICIES	**Debiasing** deliberative judgments	**Capacity** to support new activities	**Integration** into existing systems
ACCOUNTABILITY	**Transparency** and clarity of goals	**Adherence** through commitment	**Proactivity** in engagement
CULTURE WITHIN INSTITUTIONS	**Inclusion** and access	**Advocacy** at institutional levels	**Reflexivity** through reflection
EVALUATIVE AND ITERATIVE FEEDBACK	**Articulation** of diverse indicators	**Systematization** to gain consistency	**Improvement** using feedback loops

INCREASED DEPTH OF CAPABILITY

Gaining increased scalability requires moving from initial definition to deeper engagement and continual improvement

SYSTEMS-LEVEL INTEGRATION

Building consistency and resiliency into new practices requires systems-level interconnectedness

As institutions increasingly adopt new assessment principles and practices, they may strive to expand the depth of their individual capabilities and develop higher levels of system integration.

However, because institutions are naturally at different stages of readiness and evolution, there is no one-size-fits all approach and indicators of progress may not look the same.

As a result, institutions at various stages of reform may benefit from focusing on different activities:

This might mean concentrating more on:

GETTING STARTED
Acknowledging the need for change

Institutions just starting to think about research and scholarship assessment reforms may not yet be ready to begin testing new practices, and instead be primarily focused on articulating and building a case for why new assessment practices will be beneficial and **aligning on values** to support them.

They might also start by identifying and **diagnosing the nature of biases** that exist in their assessment systems, which can help institutions get more specific about what issues need to be addressed more systematically in new structures and processes.

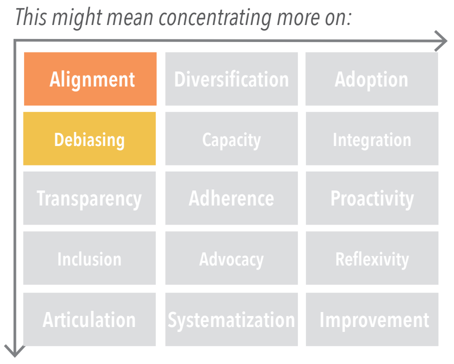

SETTING THE GROUNDWORK
Active engagement in defining new principles and practices

Research increasingly suggests that diverse groups create solutions and policies that are less biased. Actively **engaging a diverse set of participant individuals** to ensure breadth of representation can help ensure that **efforts are inclusive from the outset**, as well as contributing to more broadly applicable and relevant assessment mechanisms.

Conducting work related to assessment reform with **high levels of transparency** can also help to encourage an increased sense of credibility in the final results.

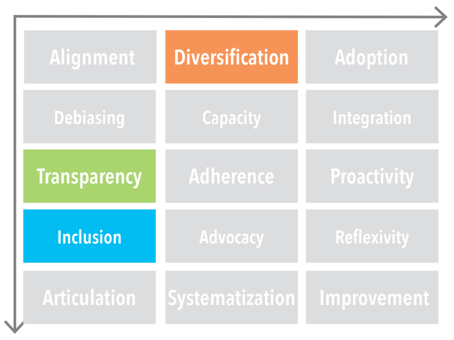

BUILDING STRUCTURAL SUPPORT
Ability, resources, and capacity to enable desired change

Ensuring that new assessment principles and practices are internalized and actively used requires addressing **issues of capacity**. This can mean setting aside sufficient time and support to learn new mechanisms or processes, but also recognizing that more holistic and qualitative inputs may initially require more processing time than metrics like JIF.

Supporting uptake may benefit from **top-down advocacy** and **structures to encourage adherence** and reduce reactance, as well as **articulating and adopting a well-rounded set of leading and lagging indicators** to more quickly identify what is working or not.

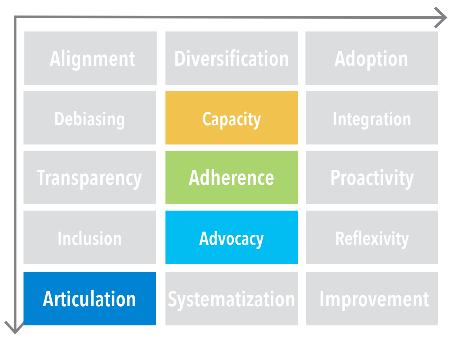

PLANNING FOR SCALE
Adoption of new assessment practices

While internalizing new principles and practices at an individual level is important, mid- or late-stage reform institutions can **increase adoption** by intentionally building in apparatuses to **systematically monitor and scale new models.**

Integrating values and desirable actions into processes and structures can increase the likelihood that new reforms are applied consistently, and can also reduce the pressure on individuals to teach or convince others given that preferred behaviors are essentially "baked in" to institutional norms and activities.

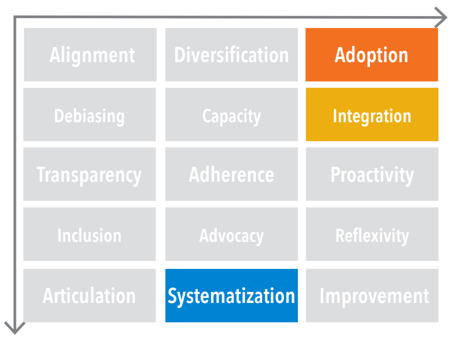

CONTINUAL IMPROVEMENT
Adaptation and refinement

Institutions at later stages of research and scholarship assessment reform will benefit from recognizing that it is an **ongoing process of monitoring and reflexivity** rather than a one-and-done accomplishment.

This requires **proactively identifying issues as conditions change**. It also means adopting an **anticipatory mindset for improvement** to recognize how success can also lead to unintended consequences, such as systems that achieve higher equity of applicants and hires but which fail to provide support post-hire mentoring or access to opportunities.

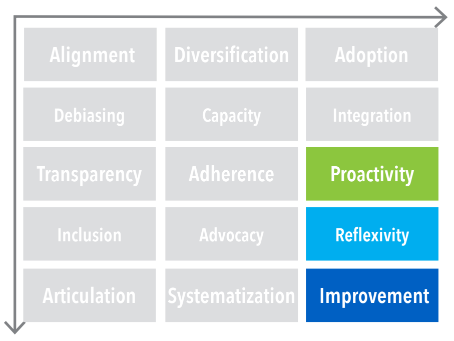

This is Open Access content distributed in accordance with the Creative Commons Attribution 4.0 International License

Hatch, A and R. Schmidt. (2021) Rethinking Research Assessment: SPACE to Evolve Academic Assessment. DORA.
